# Supplementary material for: Allosteric regulation and crystallographic fragment screening of SARS-CoV-2 NSP15 endoribonuclease
Source: Nucleic Acids Res. 2023 Apr 28;51(10):5255–70. doi: 10.1093/nar/gkad314 (PMC10250223; doi:10.1093/nar/gkad314)
Supplement: gkad314_Supplemental_Files [file gkad314_supplemental_files.zip › Supplementary datasets.zip!/81/Refine_9/01-BUSTER/Cycle-4/shell.01/R_Rfree.html]

# Rxpct factors in resolution bins at cycle 0


|  |  |  |  |  |  |  |  |  |
| --- | --- | --- | --- | --- | --- | --- | --- | --- |
|  | **<d> (Å)**  Rxpct\_Work | Rxpct\_Free | Rxpct\_All | |  |  |  |  |
|  | 15.0454 | 0.2855 | 0.2242 | 0.2832 |  |
|  | 8.6865 | 0.2163 | 0.2344 | 0.2174 |  |
|  | 6.7285 | 0.2687 | 0.2431 | 0.2672 |  |
|  | 5.6866 | 0.2931 | 0.2982 | 0.2933 |  |
|  | 5.0151 | 0.2387 | 0.2601 | 0.2400 |  |
|  | 4.5364 | 0.2244 | 0.2617 | 0.2262 |  |
|  | 4.1728 | 0.2402 | 0.2152 | 0.2388 |  |
|  | 3.8847 | 0.2627 | 0.2452 | 0.2619 |  |
|  | 3.6491 | 0.2703 | 0.2469 | 0.2692 |  |
|  | 3.4517 | 0.2752 | 0.2643 | 0.2747 |  |
|  | 3.2832 | 0.2773 | 0.2987 | 0.2787 |  |
|  | 3.1372 | 0.2677 | 0.2659 | 0.2677 |  |
|  | 3.0091 | 0.2551 | 0.2649 | 0.2555 |  |
|  | 2.8955 | 0.2410 | 0.2450 | 0.2412 |  |
|  | 2.7939 | 0.2262 | 0.2117 | 0.2255 |  |
|  | 2.7022 | 0.2061 | 0.2029 | 0.2059 |  |
|  | 2.6191 | 0.1863 | 0.2043 | 0.1874 |  |
|  | 2.5431 | 0.1786 | 0.1808 | 0.1787 |  |
|  | 2.4735 | 0.1691 | 0.1618 | 0.1687 |  |
|  | 2.4092 | 0.1751 | 0.1661 | 0.1747 |  |
|  | **Overall** | 0.2474 | 0.2443 | 0.2472 |  |

# Rxpct factors in resolution bins at cycle 1


|  |  |  |  |  |  |  |  |  |
| --- | --- | --- | --- | --- | --- | --- | --- | --- |
|  | **<d> (Å)**  Rxpct\_Work | Rxpct\_Free | Rxpct\_All | |  |  |  |  |
|  | 15.0454 | 0.2858 | 0.2242 | 0.2835 |  |
|  | 8.6865 | 0.2163 | 0.2347 | 0.2173 |  |
|  | 6.7285 | 0.2686 | 0.2431 | 0.2671 |  |
|  | 5.6866 | 0.2930 | 0.2983 | 0.2931 |  |
|  | 5.0151 | 0.2385 | 0.2604 | 0.2399 |  |
|  | 4.5364 | 0.2242 | 0.2618 | 0.2260 |  |
|  | 4.1728 | 0.2396 | 0.2147 | 0.2383 |  |
|  | 3.8847 | 0.2619 | 0.2449 | 0.2611 |  |
|  | 3.6491 | 0.2695 | 0.2467 | 0.2683 |  |
|  | 3.4517 | 0.2742 | 0.2637 | 0.2737 |  |
|  | 3.2832 | 0.2761 | 0.2980 | 0.2775 |  |
|  | 3.1372 | 0.2665 | 0.2653 | 0.2665 |  |
|  | 3.0091 | 0.2540 | 0.2645 | 0.2544 |  |
|  | 2.8955 | 0.2402 | 0.2443 | 0.2404 |  |
|  | 2.7939 | 0.2255 | 0.2115 | 0.2249 |  |
|  | 2.7022 | 0.2055 | 0.2029 | 0.2054 |  |
|  | 2.6191 | 0.1861 | 0.2044 | 0.1871 |  |
|  | 2.5431 | 0.1783 | 0.1806 | 0.1784 |  |
|  | 2.4735 | 0.1692 | 0.1617 | 0.1688 |  |
|  | 2.4092 | 0.1752 | 0.1666 | 0.1748 |  |
|  | **Overall** | 0.2469 | 0.2441 | 0.2468 |  |

# Rxpct factors in resolution bins at cycle 2


|  |  |  |  |  |  |  |  |  |
| --- | --- | --- | --- | --- | --- | --- | --- | --- |
|  | **<d> (Å)**  Rxpct\_Work | Rxpct\_Free | Rxpct\_All | |  |  |  |  |
|  | 15.0454 | 0.2859 | 0.2242 | 0.2836 |  |
|  | 8.6865 | 0.2161 | 0.2348 | 0.2172 |  |
|  | 6.7285 | 0.2682 | 0.2428 | 0.2667 |  |
|  | 5.6866 | 0.2926 | 0.2981 | 0.2928 |  |
|  | 5.0151 | 0.2382 | 0.2606 | 0.2396 |  |
|  | 4.5364 | 0.2238 | 0.2618 | 0.2257 |  |
|  | 4.1728 | 0.2390 | 0.2143 | 0.2377 |  |
|  | 3.8847 | 0.2611 | 0.2445 | 0.2603 |  |
|  | 3.6491 | 0.2686 | 0.2467 | 0.2676 |  |
|  | 3.4517 | 0.2733 | 0.2632 | 0.2728 |  |
|  | 3.2832 | 0.2751 | 0.2974 | 0.2765 |  |
|  | 3.1372 | 0.2656 | 0.2649 | 0.2655 |  |
|  | 3.0091 | 0.2532 | 0.2641 | 0.2537 |  |
|  | 2.8955 | 0.2395 | 0.2438 | 0.2397 |  |
|  | 2.7939 | 0.2251 | 0.2109 | 0.2244 |  |
|  | 2.7022 | 0.2052 | 0.2031 | 0.2051 |  |
|  | 2.6191 | 0.1859 | 0.2048 | 0.1870 |  |
|  | 2.5431 | 0.1782 | 0.1810 | 0.1784 |  |
|  | 2.4735 | 0.1693 | 0.1616 | 0.1689 |  |
|  | 2.4092 | 0.1753 | 0.1668 | 0.1749 |  |
|  | **Overall** | 0.2464 | 0.2440 | 0.2463 |  |

# Rxpct factors in resolution bins at cycle 3


|  |  |  |  |  |  |  |  |  |
| --- | --- | --- | --- | --- | --- | --- | --- | --- |
|  | **<d> (Å)**  Rxpct\_Work | Rxpct\_Free | Rxpct\_All | |  |  |  |  |
|  | 15.0454 | 0.2860 | 0.2076 | 0.2831 |  |
|  | 8.6865 | 0.2084 | 0.2513 | 0.2109 |  |
|  | 6.7285 | 0.2471 | 0.2312 | 0.2462 |  |
|  | 5.6866 | 0.2654 | 0.2493 | 0.2649 |  |
|  | 5.0151 | 0.2195 | 0.2391 | 0.2207 |  |
|  | 4.5364 | 0.2063 | 0.2404 | 0.2080 |  |
|  | 4.1728 | 0.2193 | 0.1971 | 0.2181 |  |
|  | 3.8847 | 0.2352 | 0.2277 | 0.2349 |  |
|  | 3.6491 | 0.2404 | 0.2433 | 0.2406 |  |
|  | 3.4517 | 0.2488 | 0.2474 | 0.2487 |  |
|  | 3.2832 | 0.2479 | 0.2800 | 0.2500 |  |
|  | 3.1372 | 0.2418 | 0.2574 | 0.2426 |  |
|  | 3.0091 | 0.2386 | 0.2540 | 0.2392 |  |
|  | 2.8955 | 0.2263 | 0.2372 | 0.2268 |  |
|  | 2.7939 | 0.2169 | 0.1997 | 0.2161 |  |
|  | 2.7022 | 0.1999 | 0.2171 | 0.2008 |  |
|  | 2.6191 | 0.1841 | 0.2165 | 0.1860 |  |
|  | 2.5431 | 0.1811 | 0.1912 | 0.1817 |  |
|  | 2.4735 | 0.1746 | 0.1714 | 0.1745 |  |
|  | 2.4092 | 0.1828 | 0.1784 | 0.1826 |  |
|  | **Overall** | 0.2293 | 0.2332 | 0.2295 |  |
